# Estimation of the Impact of Foodborne Salmonellosis on Consumer Well-Being in Hungary

**DOI:** 10.3390/ijerph181910131

**Published:** 2021-09-27

**Authors:** Ágnes Vajda, László Ózsvári, Dávid Szakos, Gyula Kasza

**Affiliations:** 1 Department of Veterinary Forensics and Economics, University of Veterinary Medicine Budapest, 1078 Budapest, Hungary; vajda.agnes@univet.hu (Á.V.); szakos.david@univet.hu (D.S.); 2 National Food Chain Safety Office, 1024 Budapest, Hungary; kaszagy@nebih.gov.hu

**Keywords:** salmonellosis, foodborne illness, cost of illness, WTP, consumer well-being

## Abstract

In Hungary, salmonellosis is one of the most frequent foodborne illnesses. According to our estimation, based on a representative consumer survey with 1001 respondents, the annual number of salmonellosis cases exceeded 90,000, which was 18 times higher than the officially reported data. Salmonellosis infections impose significant direct and indirect costs to the health care system, to companies (as employers) and to households. This study focused on the cost to households by analysing well-being losses due to *Salmonella* infections, for which the WTP (willingness-to-pay) method was used. WTP measures the cost that an individual would pay to avoid an undesirable harm or health outcome. For estimating WTP, 456 respondents gave quantifiable answers. The average WTP to avoid salmonellosis was 86.3 EUR. Based on this data, the total consumer well-being loss could be estimated to be about 7.87 million EUR per year in Hungary. These results indicate that consumers’ well-being losses alone would necessitate further interventions for *Salmonella* reduction.

## 1. Introduction

Salmonellosis is one of the most commonly reported zoonoses in humans in the European Union [1]. Most cases are caused by the *S.* Enteritidis (60.9%) and *S.* Typhimurium (13.8%) subtypes. *Salmonella* infections can be divided into two major groups: typhoid-type diseases (caused by *Salmonella* Typhi and *Salmonella* Paratyphi A, B, C) and nontyphoid gastrointestinal diseases (caused by other *Salmonella* strains) [2]. Symptoms of human *Salmonella* infection generally include diarrhoea, fever, stomach cramps, sometimes nausea, vomiting, bloody diarrhoea and headache [3,4]. Sources of infections for humans include domestic animals and contaminated food (primarily poultry meat, eggs and milk products), but also interaction with infected persons and noncompliance with hygiene rules [2,4]. Among the risk factors, consumer behaviour in households, which typically springs from knowledge deficiency and inconsistent kitchen practices, has a significant role [5,6,7,8,9,10].

According to statistics, salmonellosis is one of the most frequent foodborne illnesses in Hungary and is among the nearly 70 notifiable diseases [11]. Based on data from 2014 and 2018, Hungarian rates of confirmed *Salmonella* cases were more than two times higher than the EU average (53.1 and 42.6 vs. 20.7 and 20.1 per 100,000) [1]. It should be emphasised that the true occurrence of salmonellosis cases may be several times higher than that registered in the national and international databases. The number of cases in a given country can be estimated on the comparison of registered and measured cases. The number obtained this way is a country- and pathogen-specific multiplier, which was estimated to 66.8 (10.2–199.1, CI 95%) for Hungary according to a stochastic model study [12].

*Salmonella* infections represent significant cost for households, the health care system and companies (because of absent employees). The cost of the disease is often divided into two categories: direct and indirect costs. Both cost categories place a considerable burden on affected households and on society (Table 1) [13,14].

Direct costs include costs such as cost of treatment and medication at home and in hospitals, while indirect costs include additional costs due to altered consumer behaviour as well as loss due to pain and other psychological suffering.

Lack of confidence in certain products due to food contamination can deliver disastrous economic effects at the social level. There are already several methods to assess the impact of foodborne events. Willingness-to-pay (WTP) analysis is among the most frequently used methods to identify the maximum price a buyer is willing to pay for a given quantity of a product [15,16]. In the case of food safety, it can be used to measure the willingness of consumers to pay to reduce the risk of becoming ill because of food consumption [17]. The WTP method, combined with sociodemographic parameters and information on the attitudes of survey respondents, allow analysis of the impact of different personal attributes and knowledge [18].

The WHO (World Health Organisation) defines well-being as part of health. However, well-being sits outside the medical model of health, as its presence or absence is not a diagnosis. Perception of well-being is subjective and varies from individual to individual; therefore, it cannot be easily defined or measured [19]. When estimating the well-being effects of nonmarket goods, economists often rely on information about individuals’ WTP [20]. Consequently, WTP can be seen as a tool that helps to understand the value of well-being losses.

## 2. Materials and Methods

### 2.1. Data Collection and Sociodemographic Characteristics of the Samples

In our work, two different sets of data were used. Both datasets derived from the annual quantitative consumer survey of the Hungarian National Food Chain Safety Office, which has been conducted since 2012 with a standardised personal sampling methodology. To estimate the occurrence of salmonellosis, data from the 2017 survey (*n* = 1001) were used. For WTP calculation, data from the 2019 survey (*n* = 1001) were used. Both samples were representative in terms of age, sex and geographic distribution on the NUTS-2 level of the total adult population of Hungary, based on the latest census (Table 2).

The invariable parts of the NFCSO’s questionnaires—which include the demographic background variables—were designed by a group of experts including sociologists, food safety experts and consumer research specialists according to the experience in quantitative studies in the field of food safety [22,23,24].

During the data collection, respondents were informed about the aim of the research and the data management principles of the study. If the respondents were willing to participate, the quota parameters (age, sex, geographical location) were recorded, which allowed the quota numbers to be tracked by the interviewers to ensure an appropriate level of representability [25,26,27].

For WTP calculations, 456 quantifiable answers were received for the open question. Responses above 1536.6 EUR (500,000 HUF) were considered outliers and excluded from the analysis (the HUF/EUR exchange rate from 2019 was used [28]). Table 3 shows the sociodemographic characteristics of the subsample that was used for the WTP calculations.

### 2.2. Multiplier Calculation

In the estimation of the ratio of salmonellosis cases, respondents had to answer some specific questions, such as “Did you suffer from disease due to food consumption in the past year?” and “Did you suffer from fever, diarrhoea or/and vomiting in the past year?” Depending on the answers given to the aforementioned questions, respondents were asked about the suspected causes and sources of their illnesses. Case definitions of fever (elevated body temperature above 38 °C) and diarrhoea (three or more loose liquid bowel movements per day) were also given in the survey [29,30]. In order to answer questions relating to previously experienced health problems, the following answers were selectable: “Yes/No”, “Not certain” or “I do not remember”. For estimating the ratio of true occurrences and registered salmonellosis cases, only “Yes” answers were taken into consideration. According to scientific literature, nontyphoid Salmonella infections seem to be the most frequent cause of diarrhoeal disease in the European region [31]. As neither the source nor the causing organisms were shown in national epidemiological reports, all cases were considered nontyphoidal.

### 2.3. Estimation of Willingness to Pay

WTP analysis was based on the following open-ended question: “How much would you pay to avoid a Salmonella infection? (Salmonellosis is an infection, which generally causes diarrhoea, cramps, shivering and relatively high fever with a recovery time of 3–4 days.)”.

For statistical evaluation, IBM SPSS 22.0 was used. To assess the impact of demographic parameters (e.g., sex, age, educational level, economic status, residence) on WTP, the Kruskal–Wallis nonparametric test (CI 95%), which is commonly applied when the distribution of the measurement variable is not normal and does not meet the criteria for one-way ANOVA, was used [32].

## 3. Results

### 3.1. Estimated Number of Foodborne Salmonellosis Cases

Out of the 1001 interviewed participants, 245 stated that they were affected by diarrhoeal disease in the year before the survey (in 2016). Eighty-five persons experienced foodborne disease symptoms after food consumption. In the estimation of the number of salmonellosis cases, we compared the results of our survey with data from national epidemiological reports. As detailed information on infectious diseases was available only until 2015, we used this data (5069 cases of salmonellosis were then registered in the national database) as a basis for the calculation [33]. This was equal to 0.05% of the total population, which was 9,830,485 in 2015 [21]. 

**Figure 1:**
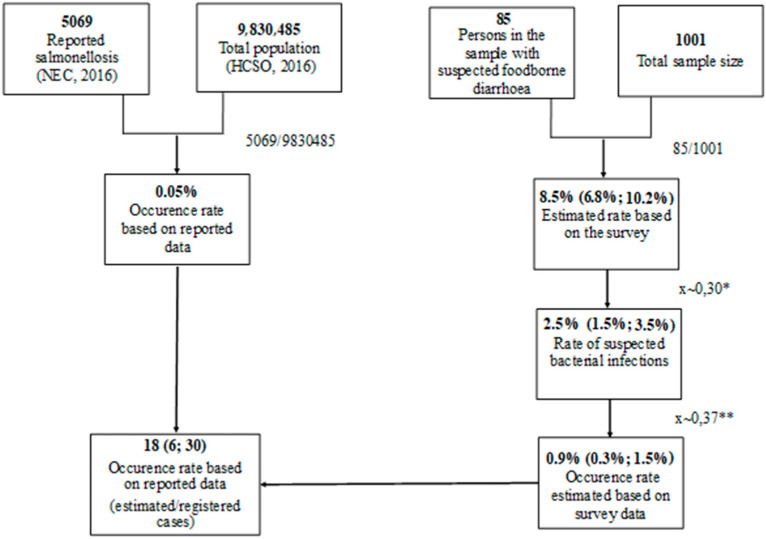
Estimation of the occurrence of foodborne salmonellosis cases based on comparison of survey results to the reported cases. * According to international literature, approximately 30% of food-related diseases are bacterial pathogens [34,35]. ** Based on national statistics, it was assumed that the ratio of salmonellosis among all bacterial infections was 37% (5069 out of 13,703 cases) [33].

Based on the survey results, 8.5% (6.8–10.2%, CI 95%) of the respondents thought their health problems were related to food consumption. Hungarian data was not available about the proportion of bacterial cases; however, approximately 30% of enteric foodborne disease is bacterial according to literature [34,35]. Consequently, 2.5% (1.5–3.5%; CI 95%) of the respondents may have suffered from bacterial foodborne disease. According to the national statistics, it was assumed that the ratio of salmonellosis among all bacterial infections was 37% (5069 out of 13,703 cases) [33]. Thus, the occurrence of salmonellosis was estimated to be 0.9% (0.3–1.5%; CI 95%) in the Hungarian population. To conclude, the occurrence of foodborne salmonellosis was approximately 18 (6–30; CI 95%) times higher than registered in the national statistics (Figure 1).

### 3.2. Hungarian Consumer WTP for Avoiding Foodborne Salmonellosis

Our results showed that the mean of consumer WTP for avoiding *Salmonella* infection was 86.3 EUR (28,067 HUF). Both the median and mode were 30.7 EUR (10,000 HUF). Out of 456 respondents, 59 rejected the idea of paying to avoid risks originating in food consumption. By contrast, 10.1% of the sample (46 out of 456 individuals) said they would pay 153.8–461.0 EUR (50,000–150,000 HUF) (Table 4).

Salmonellosis occurrence data from 2019 (5173 registered cases) [36], multiplied by the calculated multiplier (18) and the WTP value, led to the estimation that consumers’ well-being loss was 7.87 million EUR ((5173 × 18 × 28,067)/325.4, where 325.4 was the exchange rate of HUF/EUR in 2019).

### 3.3. Relationship between Demographic Parameters and WTP

Results obtained from the Kruskal–Wallis test showed statistically significant differences between respondents depending on their age (*p* < 0.0001), economic status (*p* < 0.0001), level of income (*p* < 0.0001) and level of education (*p* = 0.05). Statistically significant differences were also found for persons who lived in different regions (*p* = 0.01) and lived without or with one or more children under the age of 15 (*p* = 0.01) (Table 5).

## 4. Discussion

There have already been several studies dealing with the estimation of the real case numbers of different foodborne diseases. However, the number of those investigating the probable incidence and the economic effect of these diseases at the same time is relatively low. In Hungary, the economic impact of these diseases has been addressed in only a few studies so far [14]. Previous research was based on registered data originating from national or international databases and examined costs and outcomes from a different perspective. As an example, Országh et al. [37] examined the cost-utility of the nontyphoidal *Salmonella* control program from the state’s perspective.

The estimated annual number of salmonellosis cases was found to be well above 90,000 in Hungary. The multiplier created to calculate the true occurrence was 18 (6–30). Similar research that focused on Hungary could not be found, but international studies used 66.8 multiplier for Hungary based on the risk of illness in returning Swedish travellers for the period 1997–2003 [12,38,39]. According to a report by the EFSA, EU countries have made considerable investments in *Salmonella* control programmes; thus, the reported incidence of salmonellosis has decreased significantly [40].

We also found that the mean value of consumer WTP for avoiding foodborne salmonellosis was 86.3 EUR (28,067 HUF) in Hungary. This number was influenced significantly by the type of residence and economic status of the respondents. Considering the confirmed salmonellosis incidence in 2015 (reference year for the estimation) and the multiplier (18; 6–30; CI 95%) calculated in this study, the consumer’s well-being loss was estimated to be 7.87 million EUR (2.6–13.1 million; CI 95%) annually in Hungary. 

The ratio of well-being costs can be as much as 84.1% within the salmonellosis-related costs of the households based on a recent Hungarian study [41]. This study revealed that besides of the costs for households (14.8% of the total economic burden), the majority of social loss appears in the business sector (84.5%) because of the decline in productivity caused by the lost working days. Extra costs that appeared in the health care system amounted to only 1% of the total social burden.

## 5. Conclusions

Our findings indicate that the impact of nontyphoidal *Salmonella* infections on consumers’ well-being is very significant. Considering that other foodborne pathogens are also likely to cause serious well-being loss for the population, the authors call attention to the necessity of developing new preventive programs in the area of food safety. The calculation of the probable number of nontyphoidal salmonellosis cases and the estimation of consumers’ WTP presented in this paper can foster further research and support policy making. Policy makers traditionally consider reported incidence and direct costs when evaluating cost–benefit ratios of interventions, while nonreported cases and indirect costs account for the majority of the total social burden in case of foodborne illnesses. This paper and similar studies might help in quantifying these factors, which would in turn help optimise governmental measures.

## Figures and Tables

**Table 1:** Costs associated with salmonellosis at social level.

Public Sector Costs	
**Costs of Health Care**	**Costs of Investigation and Testing**
–Physician visits	–Disease surveillance cost
–Laboratory cost	–Research
–Hospitalisation, medications	–Outbreak cost
–Ambulance or travel cost	–Other considerations
**Costs of Households and Society**	
–Direct cost of illness to households (e.g., caretaker for ill person)	
–Pain and other psychological suffering, risk aversion	
–Cost attributable to loss of productive activity	
–Cost attributable to pain, suffering and death	
–Cost to industry (e.g., extra cleaning/cooking time cost; extra cost of refrigerator, freezer, etc.)	
–Increased food cost (willingness to pay for more expensive but safer food), etc.	

Source: Own compilation based on [13,14].

**Table 2:** Representative sociodemographic characteristics of the samples (%).

Sociodemographic Categories		Sample 2017 (*n* = 1001)	Sample 2019 (*n* = 1001)	Population *
Sex	Female	52.55	53.45	53.07
	Male	47.45	46.55	46.93
Age group	18–29	16.68	17.88	17.59
	30–39	19.78	16.88	17.04
	40–59	34.07	33.57	33.83
	>60	29.46	31.67	31.54
Geographical distribution (NUTS-2)	Central Hungary	30.07	31.17	30.75
	Central Transdanubia	11.49	11.09	10.80
	Western Transdanubia	10.59	10.39	10.03
	Southern Transdanubia	8.69	8.29	9.13
	Northern Hungary	11.99	11.79	11.62
	Northern Great Plain	14.88	14.58	14.90
	Southern Great Plain	12.29	12.69	12.78

* Latest census data of Hungarian Central Statistical Office of adult Hungarian population [21].

**Table 3:** Sociodemographic characteristics of the WTP sample, %.

Sociodemographic Categories		WTP Sample (*n* = 456)
Sex	Female	53.8
	Male	46.2
Age group	18–44	57.5
	45–64	27.9
	>65	14.9
Geographical distribution (NUTS-2)	Central Hungary	31.2
	Central Transdanubia	10.4
	Western Transdanubia	11.9
	Southern Transdanubia	9.7
	Northern Hungary	10.0
	Northern Great Plain	12.6
	Southern Great Plain	14.2
Type of residence	Capital (Budapest)	22.8
	City	64.0
	Village	13.2
Number of persons in the household	1 person	13.2
	2 persons	32.4
	3 persons	21.5
	4 persons	21.5
	>4 persons	11.4
Children under the age of 15 in the household	Yes	23.4
	No	76.6
Responsible for food shopping	Respondent themselves	35.6
	Together with a family member	53.0
	Other person	11.4
Level of income	Low	0.7
	Below average	12.1
	Average	61.2
	Above average	24.3
	High	1.8
Economic status	Employeed	55.4
	Self-employed	7.0
	Pensioner, disability pensoner	17.5
	Job seeker	0.7
	Homemaker	1.3
	Student	18.2
Level of education	Primary school	2.2
	Vocational school	5.1
	Secondary school	33.5
	University, college	59.2
Scientific studies/background	Yes	26.0
	No	74.0
Occupation related to food production	Yes	12.8
	No	87.2
Special diet	Yes	19.8
	No	80.2

**Table 4:** Consumer willingness to pay for avoiding a foodborne salmonellosis.

WTP Answers, HUF (EUR)	Number of Respondents	Percentage (%)
250,001–500,000 (768.4–1536.6)	7	1.5
200,001–250,000 (614.7–768.3)	1	0.2
150,001–200,000 (461.1–614.6)	1	0.2
50,001–150,000 (153.8–461.0)	46	10.1
20,001–50,000 (6.9–153.7)	59	12.9
10,001–20,000 (30.8–61.8)	58	12.7
1-10,000 (<30.7)	225	49.3
0	59	12.9
	456	100.0

**Table 5:** Differences in WTP answers among groups by demographic parameters.

Sociodemographic Categories		WTP Mean Values (HUF)	*p*	df	H
Sex	Female	23,789	0.26	1	1.28
	Male	32,208			
Age group	18–44	32,279	<0.0001	3	15.73
	45–64	26,545			
	>65	18,557			
Geographical distribution (NUTS-2)	Central Hungary	41,092	0.01	6	16.27
	Central Transdanubia	23,183			
	Western Transdanubia	19,810			
	Southern Transdanubia	24,789			
	Northern Hungary	10,456			
	Northern Great Plain	32,568			
	Southern Great Plain	22,305			
Type of residence	Capital (Budapest)	15,321	0.21	2	3.17
	City	26,663			
	Village	39,462			
Number of persons in the household	1 person	20,941	0.08	7	12.79
	2 persons	31,034			
	3 persons	26,648			
	4 persons	32,112			
	>4 persons	25,504			
Children under the age of 15 in the household	Yes	36,987	0.01	1	6.51
	No	25,502			
Responsible for food shopping	Respondent themselves	21,962	0.38	2	1.95
	Together with a family member	33,058			
	Other person	26,760			
Level of income	Low	1,667	<0.0001	4	39.04
	Below average	11,822			
	Average	24,755			
	Above average	36,599			
	High	96,250			
Economic status	Employeed	33,436	<0.0001	5	18.51
	Self-employed	36,623			
	Pensioner, disability pensoner	18,591			
	Job seeker	41,667			
	Homemaker	22,083			
	Student	20,665			
Level of education	Primary school	5160	0.05	3	8.04
	Vocational school	22,596			
	Secondary school	24,147			
	University, college	28,393			
Scientific studies/background	Yes	37,175	0.07	1	3.32
	No	30,667			
Occupation related to food production	Yes	20,556	0.75	1	0.10
	No	29,778			
Special diet	Yes	26,880	0.15	1	2.06
	No	28,906			

## Data Availability

The data presented in this study are available on request from the corresponding author without undue reservation.
